# Tph Cells Expanded in Primary Sjögren’s Syndrome

**DOI:** 10.3389/fmed.2022.900349

**Published:** 2022-06-09

**Authors:** Weiqian Chen, Fan Yang, Jin Lin

**Affiliations:** ^1^Division of Rheumatology, The First Affiliated Hospital, Zhejiang University School of Medicine, Hangzhou, China; ^2^State Key Laboratory of Infectious Diseases Diagnosis and Treatment, School of Medicine First Affiliated Hospital, Zhejiang University School of Medicine, Hangzhou, China

**Keywords:** primary Sjögren’s syndrome, PD-1, CXCR5, ESSDAI score, Tph cells

## Abstract

**Objectives:**

PD-1^+^CXCR5^–^CD4^+^T peripheral helper cells, named Tph cells, contribute to B-cell immune responses and the production of antibodies in systemic lupus erythematosus and rheumatoid arthritis. However, the role of Tph cells was unknown in the pathogenesis of primary Sjögren’s syndrome (pSS). Here, we aim to explore the contribution of Tph cells in the development of pSS.

**Methods:**

Sixty patients with pSS and 61 age and sex-matched healthy individuals were recruited for this study. The frequency of Tph cells in the blood was measured by flow cytometry. The expression of inducible T-cell costimulator (ICOS), MHC-II, IL-21, CCR2, CCR5, and CCR9 was evaluated in Tph cells. The relationship between Tph cells and indicators of clinical disease was assessed. Co-expression levels of PD-1, CXCR5, CD4, CCR2, and CCR5 in the salivary gland specimens from patients with pSS and patients with dry mouth and eyes but normal pathology were also analyzed.

**Results:**

We demonstrated increased circulating Tph cells (7.53 ± 6.65% *vs.* 3.08 ± 1.31%, *p* < 0.0001) in patients with pSS (*n* = 60) compared to healthy controls (*n* = 61). Tph cells were significantly associated with the ESSDAI disease activity scores, IgG, ESR, IL-21, anti-SSA antibody, and CD138^+^/CD19^+^ plasma cells. Furthermore, ICOS was highly expressed in Tfh and Tph cells in patients with pSS. IL-21, MHC-II, CCR2, and CCR5 expression was higher in pSS Tph cells, and CCR9 expression was lower in pSS Tph cells than in pSS Tfh cells. Moreover, Tph cells and CCR2^+^CD4^+^T and CCR5^+^CD4^+^T cells were found in the labial gland of patients with pSS.

**Conclusion:**

Our data show that Tph cells were enriched in peripheral blood and labial gland of patients with pSS. Circulating Tph cells correlated with disease activity scores, suggesting a crucial role of Tph in the development of pSS.

## Introduction

Primary Sjögren’s syndrome (pSS) is a diffuse connective tissue disease characterized by a high degree of lymphocytic infiltration in the lacrimal and salivary glands. Patients with pSS may have xerostomia, xerophthalmia, and extra-glandular manifestations. The precise etiopathogenesis of pSS remains unclear. The hyperactivation of B cells is a hallmark feature of pSS, characterized by high titers of anti-SSA and anti-SSB autoantibodies, rheumatoid factor, hyperglobulinemia, and a high risk of B-cell lymphoma (1, 2). Expanded plasmablasts, plasma cells, memory B cells, and marginal zone (MZ) B cells are the key B-cell subsets that exist in patients with pSS (3). Autoreactive T-B cell interactions promote B-cell activation in lymphoid tissue, contributing to the production of autoantibodies by plasma cells in pSS (4). Follicular helper T cells (Tfh) are a specialized subset of CD4^+^ T cells located in B-cell follicles that express mainly CXCR5, inducible T-cell costimulator (ICOS), and programmed cell death 1 (PD-1). Tfh cells promote germinal center (GC) formation through IL-21 and CXCL13, the differentiation of GC B cells into memory B cells or plasma cells, the development of high-affinity antibodies, and the class switching of immunoglobulins to maintain a long-term humoral immune response (5). Tfh cells were increased in the peripheral blood of patients with pSS, and their frequency was correlated to the EULAR Sjögren’s syndrome disease activity index (ESSDAI) (6). The interaction between Tfh cells and GC B cells results in B-cell activation and the generation of memory B cells or plasma cells, ultimately promoting the development of pSS (6).

A recent study showed that peripheral helper T (Tph) cells are present in the synovium of patients with rheumatoid arthritis (RA), helping B-cell responses and the formation of plasma cells. Tph cells express markers similar to Tfh cells, such as ICOS, PD-1, IL-21, and CXCL13; however, they do not express CXCR5 (7). The increased circulating Tph cells were positively correlated with systemic lupus erythematosus (SLE) disease activity (8–11). Tph cells highly expressed IL-21 and ICOS, thus having the ability to regulate B-cell differentiation. c-Maf, a transcription factor downstream of IL-21 signaling, was expressed in lupus Tph cells, suggesting the importance of c-Maf in the upregulation of IL-21 expression and its participation in the pathogenesis of SLE (10). In addition, circulating Tph cells were increased in patients with IgG4-related disease (12). Circulating Tph cells have been reported to be increased in the peripheral blood of patients with pSS and are positively correlated with disease activity (1, 13, 14). Pontarini et al. found that Tph cells were enriched in the salivary gland tissue with the GC in patients with pSS (1). However, the characteristic of circulating Tph cells from patients with pSS was unclear, especially for their chemokine receptor expression. We assessed the characteristics of circulating Tph cells and labial tissue Tph cells in patients with pSS and further clarified their role in the pathogenesis of pSS.

## Materials and Methods

### Subjects and Clinical Features

This study included 60 patients with pSS from the Division of Rheumatology, the First Affiliated Hospital, Zhejiang University School of Medicine. All patients with pSS met the revised 2002 American–European criteria (15). Patients with pSS did not receive any glucocorticoids, immunosuppressive agents, or biological agents, such as Rituximab, Belimumab, and Abatacept, 3 months prior to inclusion in this study. This study was carried out from August 2018 to May 2020. The patients’ clinical data originated from electronic medical records. The current disease activity of patients with pSS was evaluated by ESSDAI scores (16). Sixty-one healthy individuals were recruited as non-autoimmune controls without cancer and infection. All participators signed written informed consent forms. The research was carried out within the framework of the Declaration of Helsinki. The Medical Ethical Committee of The First Affiliated Hospital, Zhejiang University School of Medicine, approved this study (#2017-638).

### Flow Cytometry

Peripheral blood mononuclear cells (PBMCs) were obtained from patients with pSS and the control group. For cell-surface staining, the following fluorescence-conjugated mouse anti-human antibodies were added: PerCP/Cy5.5 anti-CD4 (OKT4), PE anti-CXCR5 (J252D4), APC anti-PD-1(EH12.2H7), Brilliant Violet 510 anti-CD45RA (HI100), Brilliant Violet 421 anti-ICOS (C398.4A), FITC anti-major histocompatibility complex (MHC)-II (L243), APC/Cyanine7 anti-CCR2(FN50), FITC anti-CCR5(HEK/1/85a), PerCP/Cy5.5 anti-CCR9(L053E8), APC Cy7 anti-CD4 (OKT4), PE/Cy7 anti-CD19(HIB19), and Alexa Fluor 700 anti-CD138 (MI15), or relevant isotype controls. CD138^+^/CD19^+^ B cells were identified as plasma cells. For intracellular staining, PBMCs were stimulated by PMA and Ionomycin for 5 h and brefeldin A for the last 4 h of the stimulation. Then, the cells were stained with CD4, CXCR5, and PD-1. Next, they were fixed and permeabilized for the intracellular staining of IL-21 (3A3-N2) using BD Pharmingen Transcription Factor Buffer Set. Finally, PBMCs were detected and analyzed by using a BD LSRFortessa flow cytometer.

### Immunofluorescent Staining of Tissue

Labial gland tissue biopsy specimens were obtained from ten patients with pSS and eight individuals who suffered from dry mouth but without focal lymphocytic infiltration. Labial gland tissues specimens were placed in liquid nitrogen, embedded, and sectioned (5 μm). Then, antigen retrieval was performed, followed by blocking with serum. Next, tissue slides were incubated with rabbit anti-human PD-1, CXCR5, CCR2, or CCR5 and mouse anti-human CD4 primary antibodies. They were then washed and incubated with Alexa 488 labeled donkey anti-rabbit secondary antibody or Alexa 647 labeled donkey anti-rat secondary antibody. Finally, DAPI was added to stain nuclei. The simultaneous expression of PD-1, CXCR5, CCR2, and CCR5 in CD4^+^T cells was observed.

### Statistical Analysis

We presented the results as median and standard deviation (SD). We performed comparisons between two groups using the non-parametric Mann-Whitney test. We evaluated the correlation between the Tph cells or Tfh and PD-1^–^CXCR5^–^CD4^+^T cells and clinical data using Spearman’s coefficient of correlation. The heatmap was generated by HemI software (a heatmap illustrator) according to the correlation value. We used SPSS software, version 19.0 for statistical analysis. *P*-values < 0.05 were set as statistically significant.

## Results

### Circulating Tph Cells Were Expanded in the Peripheral Blood of Patients With pSS, and Tph Cells Expressed IL-21

**FIGURE 1:**
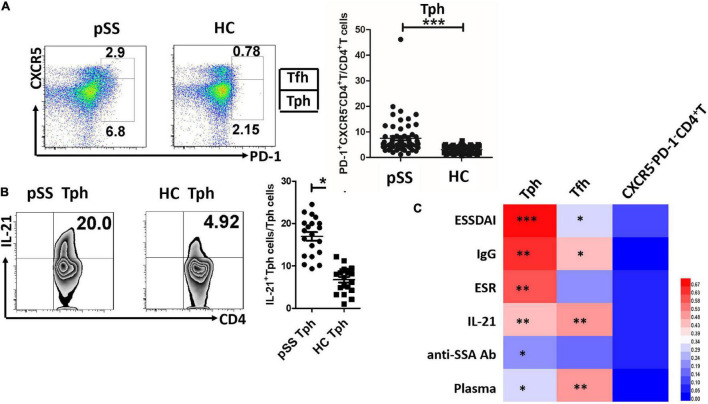
Circulating Tph cells were increased in patients with pSS, and they were associated with disease activity indexes and plasma cells. **(A)** Circulating PD-1^+^CXCR5^–^CD4^+^T cells (Tph) and PD-1^+^CXCR5^+^CD4^+^T cells (Tfh) were exhibited in one patient with primary Sjögren’s Syndrome (pSS) and one healthy control (HC). The summarized data were demonstrated in patients with pSS (*n* = 60) and HCs (*n* = 61) (***p < 0.001). **(B)** The expression of IL-21 was increased in Tph cells from patients with pSS (*n* = 20), compared to HCs (*n* = 20) (**p < 0.01). **(C)** The heatmap was generated by HemI software (a heatmap illustrator) according to the correlation value. Circulating Tph cells were significantly related to EULAR Sjögren’s syndrome disease activity index (ESSDAI) score, IgG, erythrocyte sedimentation rate (ESR), IL-21, anti-SSA antibody, and CD138^+^/CD19^+^ plasma cells (*p < 0.05, **p < 0.01, ***p < 0.001). Tfh cells were also associated with ESSDAI score, IgG, IL-21, and plasma cells. PD-1^–^CXCR5^–^CD4^+^T cells were not associated with these disease activity parameters.

Baseline data from 60 patients with pSS and 61 healthy controls are shown in Table 1. There were no significant age and sex differences between the pSS group and healthy controls. In the pSS group, the ESSDAI score ranged from 1 to 12 points, and the mean ESSDAI score was 4.5 ± 3.0. The PD-1^+^CXCR5^–^CD4^+^T cells (Tph) and PD-1^+^CXCR5^+^CD4^+^T cells (Tfh) were increased in the peripheral blood of patients with pSS compared to healthy controls (Figure 1A). Circulating Tph cells were 7.53 ± 6.65% in patients with pSS and were 3.08 ± 1.31% in healthy controls (*p* < 0.0001) (Figure 1A and Table 1). Furthermore, the expression of IL-21 was higher in Tph cells in patients with pSS than those in HCs (Figure 1B). We also found that Tfh cells expressed IL-21 in patients with pSS (Supplementary Figure 1).

**TABLE 1:** Demographic and clinical parameters of pSS and healthy controls on study entry.

	pSS (*n* = 60)	Controls (*n* = 61)	*P*-value
Age, mean ± SD years	53.5 ± 14.5	49.0 ± 15.9	0.213
Female/male	57/3	57/4	0.716
Disease duration, mean ± SD months	59.9 ± 89.5	NA	NA
Constitutional symptoms(±)	19/41	NA	NA
Lymphadenopathy(±)	25/35	NA	NA
Glandular swelling(±)	13/47	NA	NA
Arthritis (±)	12/48	NA	NA
Cutaneous involvement(±)	7/53	NA	NA
Lung involvement (±)	15/45	NA	NA
Renal involvement (±)	3/57	NA	NA
Peripheral neuropathy(±)	2/58	NA	NA
Hematologic disorder(±)	44/16	NA	NA
Biopsy focus score (number of lymphocytic foci/4 mm^2^)	2.2 ± 1.4	NA	NA
Anti-SSA(%)	42/18	NA	NA
Anti-SSB(%)	26/34	NA	NA
IgG, mg/dL	1863 ± 681.1	NA	NA
ESR, mean ± SD mm/h	36.6 ± 26.9	NA	NA
RF, mean ± SD U/L	168.8 ± 469.4	NA	NA
C3, mean ± SD mg/dl	96.6 ± 23.3	NA	NA
C4, mean ± SD mg/dl	18.5 ± 8.3	NA	NA
ESSDAI scores, mean ± SD	4.5 ± 3.0	NA	NA
PD-1^+^CXCR5^–^CD4^+^T cells (Tph)/CD4^+^T cells (%)	7.53 ± 6.65	3.08 ± 1.31	< 0.0001
PD-1^+^CXCR5^+^CD4^+^T cells (Tfh)/CD4^+^T cells (%)	3.42 ± 2.35	1.25 ± 0.56	< 0.0001

*NA: not applicable; IgG, 700-1600mg/dl; ESR: Erythrocyte sedimentation rate, normal range 0–20mm/h; RF, rheumatoid factor, normal range 0–20 U/L; C3, normal range 58–160 mg/dL; C4, normal range 7–49 mg/dL; ESSDAI, EULAR Sjögren’s syndrome disease activity index.*

### Circulating Tph Cells Were Related to Disease Activity Parameters and Plasma Cells in Patients With pSS

Like circulating Tfh cells (*r* = 0.283, *p* = 0.011), circulating Tph cells (*r* = 0.828, *p* < 0.0001) were significantly associated with the ESSDAI score (Figure 1C). Circulating Tph cells were also associated with IgG (*r* = 0.545, *p* < 0.01), the erythrocyte sedimentation rate (ESR) (*r* = 0.446, *p* < 0.01), serum IL-21 levels (*r* = 0.403, *p* < 0.01), and anti-SSA antibodies (*r* = 0.223, *p* = 0.017) (Figure 1C), but not disease course, C-reactive protein (CRP), C3, and C4. Moreover, circulating Tph cells were higher in patients with pSS with lymphadenopathy (11.06 ± 8.86% vs. 4.74 ± 2.01%, *p* = 0.002), cutaneous involvement (14.27 ± 14.91% vs. 6.52 ± 4.19%, *p* = 0.003), pulmonary involvement (10.91 ± 10.7% vs. 6.26 ± 4.16%, *p* = 0.02), hematologic disorder (neutropenia, lymphopenia, anemia, or thrombocytopenia) (8.84 ± 7.39% vs. 3.83 ± 1.80%, *p* = 0.001), and biological changes (hypocomplementemia or hypergammaglobulinemia) (8.50 ± 7.38% vs. 4.20 ± 1.84%, *p* = 0.001) (data not shown), compared to patients with pSS who had no relevant symptoms. Interestingly, circulating Tph cells were related to CD138^+^/CD19^+^ plasma cells (r = 0.324, *p* = 0.018) (Figure 1C).

### ICOS, MHC-II, CCR2, and CCR5 Expressions Were Higher in pSS Tph Cells

**FIGURE 2:**
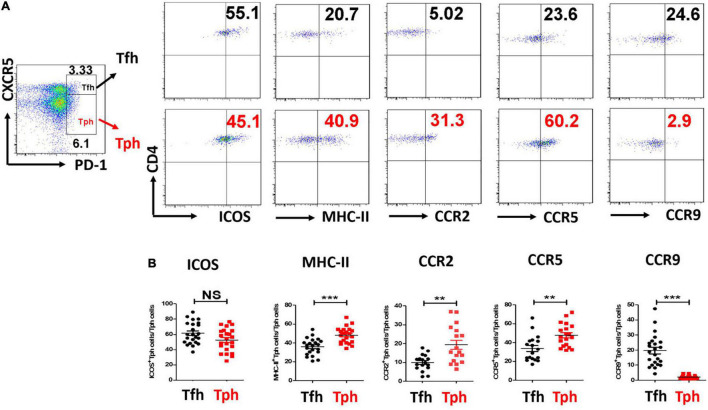
CCR2, CCR5 expressions were all higher, but CCR9 expression was lower in pSS Tph cells. **(A,B)** Inducible T-cell costimulator (ICOS), Major histocompatibility complex (MHC)-II, CCR2, CCR5, and CCR9 expressions were measured in Tph cells and Tfh cells from pSS patients. The summarized data were exhibited. (**p < 0.01, ***p < 0.001).

Furthermore, ICOS expression was similarly high in pSS Tph and Tfh cells. MHC-II, CCR2, and CCR5 expressions were higher in Tph cells than in Tfh cells from patients with pSS. CCR9 expression was lower in Tph cells from patients with pSS than in pSS Tfh cells (Figure 2). Furthermore, CCR2^+^Tph cells and CCR5^+^Tph cells were positively associated with the ESSDAI score (*r* = 0.485, *p* = 0.035; *r* = 0.538, *p* = 0.017), CCR9^+^Tph cells were negatively associated with the ESSDAI score (r = −0.458, *p* = 0.028). However, no significant association of ICOS^+^Tph cells or MHC-II^+^Tph cells with ESSDAI score.

### Tph, CCR2^+^CD4^+^T, and CCR5^+^CD4^+^T Cells Were Found in Labial Gland Tissue of Patients With pSS

**FIGURE 3:**
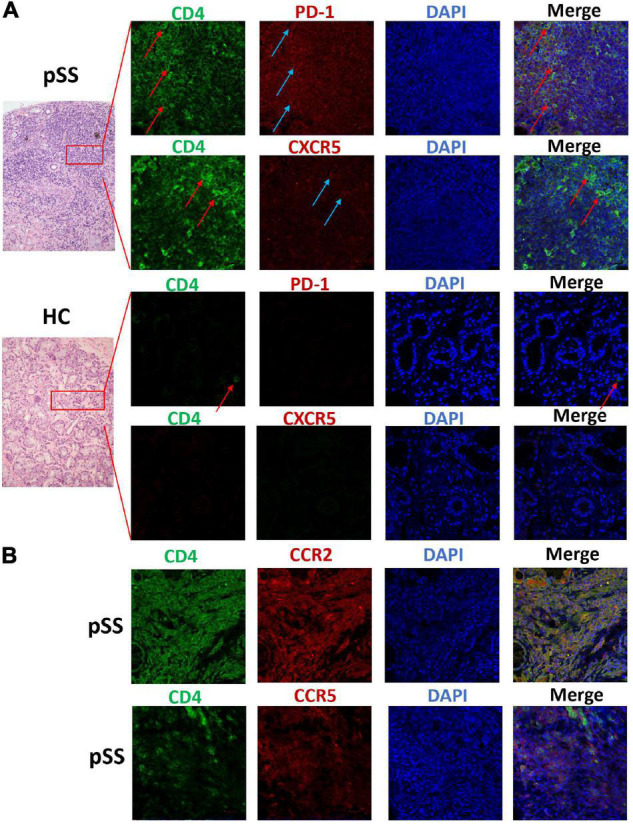
Tissue Tph cells, CCR2^+^CD4^+^T cells, and CCR5^+^CD4^+^T cells existed in the labial gland of pSS patients. We analyzed the co-expression levels of PD-1, CXCR5, CD4, CCR2, and CCR5 in the labial gland specimens of one patient with pSS and one patient with dry mouth and eyes but normal pathology. **(A)** There was a large number of CD4^+^T cells (the first and second layers, green FITC) in the infiltrating lymphocytes of the labial gland from pSS patients, and the expression of PD-1 was also significantly increased (the first layer, red Alexa Fluor 647), but CXCR5 expression was few, mostly CD4^+^T cells were negative for CXCR5 (second layer, red Alexa Fluor 647); while the control group was a dry mouth and eye patient without primary Sjögren’s syndrome, and a few CD4^+^ T cells were seen (third layer, red arrows), PD-1 and CXCR5 expression were both negative (third and fourth layers, red Alexa Fluor 647). **(B)** CCR2^+^CD4^+^T cells (layer 5) and CCR5^+^CD4^+^T cells (layer 6) were found in pSS labial gland tissue.

This study collected labial gland specimens from ten patients with pSS and eight patients with xerostomia, but no pSS. Co-expression levels of PD-1, CXCR5, CD4, CCR2, and CCR5 were analyzed in labial gland specimens by immunofluorescent staining. CD4^+^T cells infiltrated the labial gland of patients with pSS, which were PD-1 positive and mostly CXCR5 negative. CXCR5^+^PD1^+^CD4^+^ Tfh cells were also found in the labial gland of patients with pSS. This suggests that Tph cells (PD-1^+^CXCR5^–^CD4^+^T) are present in the labial gland specimens of pSS patients. Furthermore, CCR2^+^CD4^+^T cells and CCR5^+^CD4^+^T cells were found in the labial gland tissue of patients with pSS (Figure 3).

## Discussion

CD4^+^T cells are irreplaceable regulators of autoimmune diseases; however, the detailed mechanism of their function in the immunopathology of pSS is still unclear. It is well understood that PD-1 inhibits T cells through interactions with its ligands, PD-L1, and PD-L2, to maintain peripheral immune tolerance. PD-1^+^CXCR5^–^CD4^+^ Tph cells can be separated from PD-1^+^CXCR5^+^CD4^+^T cells (Tfh) based on the expression of CXCR5. Tph cells regulated B-cell responses and the differentiation of plasma cells in rheumatoid arthritis (7). In our study, we demonstrated that Tph cells are significantly increased in the peripheral blood of patients with pSS. Furthermore, the frequency of circulating Tph cells was significantly associated with pSS disease activity indicators. In addition, circulating Tph cells were associated with other disease indexes, such as the IgG, ESR levels, and anti-SSA antibody. Moreover, Tph cells were more frequent in patients with pSS with lymphadenopathy, cutaneous involvement, pulmonary involvement, hematologic disorder, or biological changes. Tph cells are expanded in patients with pSS, and are positively correlated with disease activity (13). Therefore, Tph cells may participate in the development of pSS.

Furthermore, we found that circulating Tph cells correlated with plasma cells in pSS. This result is consistent with data from another group (14). Rao et al. showed that Tph cells promoted the differentiation of plasma cells through IL-21 and SLAMF5 (7). We demonstrated that the expression of IL-21 was higher in Tph cells than in Tfh cells in patients with pSS, but ICOS was highly expressed in both pSS Tph cells and Tfh cells. Tfh cells promote B-cell proliferation, activation, and antibody production mainly through the key functional molecules, IL-21, CD40L, and ICOS (17). However, the functional molecules of Tph cells are still unclear. We demonstrated that Tph cells expressed IL-21 and ICOS in patients with pSS. Dupré et al. found that ICOS was highly expressed in pSS Tph cells (14). IL-21 is crucial in the differentiation and function of memory B cells and plasma cells (18). ICOS, a T-cell costimulatory molecule, interacts with its ligand, ICOSL, on the surface of B cells to promote T-cell proliferation and activation and B-cell differentiation (1). Furthermore, Tph cells in ectopic lymphoid structures expressed IL-21 and ICOS (1). Therefore, Tph cells may be a key T-cell subset for driving the pathogenic B-cell response in pSS, primarily through IL-21 and ICOS.

A recent paper demonstrated that Tph cells are found in SG with GCs (1). They found that CXCR5^–^CD4^+^PD1^hi^ICOS^+^Foxp3^–^ Tph cells co-expressed IL-21 and interferon-γ, but low IL-17 in parotid MALT-lymphoma (1). We also found that infiltration of CD4^+^T cells can be seen in the labial gland of patients with pSS, in which PD-1 expression is positive, but CXCR5 is mostly negative. It suggested the existence of Tph cells (PD-1^+^CXCR5^–^CD4^+^T) in the labial gland specimens of pSS patients. Meanwhile, we found that CXCR5^+^PD1^+^CD4^+^ Tfh cells were also present in the labial gland of patients with pSS. Other previous papers demonstrated the presence of CXCR5^+^CD4^+^T cells (19), CXCR5^+^IL-21^+^T cells (20), Bcl-6^+^CXCR5^+^ cells(21), and Bcl-6^+^CD3^+^T cells (22) in these locations in patients with pSS. There was a difference in glandular Tfh cells staining between our study and the above published studies.

Although Tph cells lacked CXCR5, they expressed the inflammatory chemokine receptors CCR2 and CCR5 (7). We found that CCR2 and CCR5 were more highly expressed in Tph cells in patients with pSS. Furthermore, Tph cells, CCR2^+^CD4^+^T cells, and CCR5^+^CD4^+^T cells were present in the labial gland tissue of patients with pSS. Blokland et al. demonstrated that the CCR2 ligand (CCL2) and CCR5 ligand (CCL5) are highly expressed in the labial glandular tissues of patients with pSS (23), suggesting that Tph cells may be recruited to the salivary glands through the CCR2/CCL2 or CCR5/CCL5 axes, and then participate in salivary gland lymphocyte infiltration and inflammation. The gut homing chemokine receptor, CCR9, was expressed at a lower level in Tph cells in patients with pSS. This suggested that Tph cells may not migrate to the gut through CCR9. Cosorich et al. demonstrated that CCR9^+^Tfh cells produced proinflammatory cytokines and migrated to the digestive system, where they cause an immune response (24). CCR9 may be a critical differentially expressed chemokine receptor between Tfh and Tph cells.

This study has some limitations; the *in vitro* coculture of pSS Tph cells with B cells was not completed. The key factors that drive human T-cell differentiation toward a Tph cell phenotype were not identified, and the transcriptional factors in the development of Tph cells are still unclear. Bcl-6 is a crucial gene in the formation of GCs and a key transcription factor in Tfh cells. However, it is expressed at a low level in Tph cells. Blimp-1 is highly expressed in Tph cells but expressed at a low level in Tfh cells (7). C-maf was highly expressed in both Tph and Tfh cells (10). In the future, we will identify the key transcription factors regulating Tph cells by sorting Tph cells, Tfh cells, and PD-1^–^CXCR5^–^CD4^+^T cells from peripheral blood for single-cell RNA sequencing analyses. Further studies are essential to investigate the biological functions of Tph cells in pSS.

## Conclusion

Circulating Tph cells, capable of producing IL-21, were markedly increased in patients with pSS. The frequency of circulating Tph cells was correlated with disease activity and B-cell differentiation. In addition, Tph cells were enriched in the labial gland of patients with pSS. We speculate that Tph cells may promote the pathogenic B-cell response in pSS.

## Data Availability Statement

The raw data supporting the conclusions of this article will be made available by the authors, without undue reservation.

## Ethics Statement

The studies involving human participants were reviewed and approved by the Medical Ethical Committee of the First Affiliated Hospital, Zhejiang University School of Medicine. The patients/participants provided their written informed consent to participate in this study. Written informed consent was obtained from the individual(s) for the publication of any potentially identifiable images or data included in this article.

## Author Contributions

WC and JL conceived to the study and wrote the manuscript. WC and FY performed the experiments and analyzed the data. All authors contributed to the article and approved the submitted version.

## Conflict of Interest

The authors declare that the research was conducted in the absence of any commercial or financial relationships that could be construed as a potential conflict of interest.

## Publisher’s Note

All claims expressed in this article are solely those of the authors and do not necessarily represent those of their affiliated organizations, or those of the publisher, the editors and the reviewers. Any product that may be evaluated in this article, or claim that may be made by its manufacturer, is not guaranteed or endorsed by the publisher.

## References

[1] Pontarini E Murray-Brown WJ Croia C Lucchesi D Conway J Rivellese F Unique expansion of Il-21+ Tfh and Tph cells under control of ICOS identifies Sjögren’s syndrome with ectopic germinal centres and malt lymphoma. *Ann Rheum Dis.* (2020) 79:1588–99. 10.1136/annrheumdis-2020-217646 32963045 PMC7677495

[2] Shiboski CH Shiboski SC Seror R Criswell LA Labetoulle M Lietman TM 2016 American college of rheumatology/European league against rheumatism classification criteria for primary Sjögren’s syndrome: a consensus and data-driven methodology involving three international patient cohorts. *Arthritis Rheumatol.* (2017) 69:35–45. 10.1002/art.39859 27785888 PMC5650478

[3] Nocturne G Mariette XB. Cells in the pathogenesis of primary Sjögren syndrome. *Nat Rev Rheumatol.* (2018) 14:133–45. 10.1038/nrrheum.2018.1 29416129

[4] Verstappen GM Kroese FGM Bootsma H. T cells in primary Sjogren’s syndrome: targets for early intervention. *Rheumatology.* (2019) 60:3088–98. 10.1093/rheumatology/kez004 30770920 PMC8516500

[5] Ueno H Banchereau J Vinuesa CG. Pathophysiology of T follicular helper cells in humans and mice. *Nat Immunol.* (2015) 16:142–52. 25594465 10.1038/ni.3054 PMC4459756

[6] Chen W Yang F Xu G Ma J Lin J. Follicular helper T cells and follicular regulatory T cells in the immunopathology of primary Sjögren;s syndrome. *J Leukoc Biol.* (2021) 109:437–47. 10.1002/jlb.5mr1020-057rr 33325085

[7] Rao DA Gurish MF Marshall JL Slowikowski K Fonseka CY Liu Y Pathologically expanded peripheral T helper cell subset drives B cells in rheumatoid arthritis. *Nature.* (2017) 542:110–4. 10.1038/nature20810 28150777 PMC5349321

[8] Lin J Yu Y Ma J Ren C Chen W. Pd-1+Cxcr5-Cd4^+^T cells are correlated with the severity of systemic lupus erythematosus. *Rheumatology.* (2019) 58:2188–92. 10.1093/rheumatology/kez228 31180450

[9] Makiyama A Chiba A Noto D Murayama G Yamaji K Tamura N Expanded circulating peripheral helper T cells in systemic lupus erythematosus: association with disease activity and B cell differentiation. *Rheumatology.* (2019) 58:1861–9. 10.1093/rheumatology/kez077 30879065

[10] Bocharnikov AV Keegan J Wacleche VS Cao Y Fonseka CY Wang G Pd-1hicxcr5- T peripheral helper cells promote B cell responses in lupus via MAF and Il-21. *JCI Insight.* (2019) 4:e130062. 10.1172/jci.insight.130062 31536480 PMC6824311

[11] Christophersen A Lund EG Snir O Solà E Kanduri C Dahal-Koirala S Distinct phenotype of Cd4^+^ T cells driving celiac disease identified in multiple autoimmune conditions. *Nat Med.* (2019) 25:734–7. 10.1038/s41591-019-0403-9 30911136 PMC6647859

[12] Kamekura R Yamamoto M Takano K Yabe H Ito F Ikegami I Circulating Pd-1^+^Cxcr5^–^Cd4^+^ T cells underlying the immunological mechanisms of Igg4-related disease. *Rheumatol Adv Pract.* (2018) 2:rky043. 10.1093/rap/rky043 31431980 PMC6649940

[13] Verstappen GM Meiners PM Corneth OBJ Visser A Arends S Abdulahad WH Attenuation of follicular helper T cell-dependent B cell hyperactivity by abatacept treatment in primary Sjogren’s syndrome. *Arthritis Rheumatol.* (2017) 69:1850–61. 10.1002/art.40165 28564491

[14] Dupré A Pascaud J Rivière E Paoletti A Ly B Mingueneau M Association between T follicular helper cells and T peripheral helper cells with B-cell biomarkers and disease activity in primary Sjögren syndrome. *RMD Open.* (2021) 7:e001442. 10.1136/rmdopen-2020-001442 33688082 PMC7944988

[15] Vitali C Bombardieri S Jonsson R Moutsopoulos HM Alexander EL Carsons SE Classification criteria for Sjögren’s syndrome: a revised version of the European criteria proposed by the American-European consensus group. *Ann Rheum Dis.* (2002) 61:554–8. 10.1136/ard.61.6.554 12006334 PMC1754137

[16] Seror R Ravaud P Bowman SJ Baron G Tzioufas A Theander E Eular Sjogren’s syndrome disease activity index: development of a consensus systemic disease activity index for primary Sjogren’s syndrome. *Ann Rheum Dis.* (2010) 69:1103–9. 10.1136/ard.2009.110619 19561361 PMC2937022

[17] Wan Z Lin Y Zhao Y Qi H. Tfh cells in bystander and cognate interactions with B cells. *Immunol Rev.* (2019) 288:28–36. 10.1111/imr.12747 30874359

[18] Tangye SG Ma CS. Regulation of the germinal center and humoral immunity by interleukin-21. *J Exp Med.* (2020) 217:e20191638. 10.1084/jem.20191638 31821441 PMC7037251

[19] Jin L Yu D Li X Yu N Li X Wang Y Cd4^+^Cxcr5^+^ follicular helper T cells in salivary gland promote B cells maturation in patients with primary Sjogren’s syndrome. *Int J Clin Exp Pathol.* (2014) 7:1988–96. 24966908 PMC4069915

[20] Kang KY Kim HO Kwok SK Ju JH Park KS Sun DI Impact of interleukin-21 in the pathogenesis of primary Sjögren’s syndrome: increased serum levels of interleukin-21 and its expression in the labial salivary glands. *Arthritis Res Ther.* (2011) 13:R179. 10.1186/ar3504 22030011 PMC3308114

[21] Maehara T Moriyama M Hayashida JN Tanaka A Shinozaki S Kubo Y Selective localization of T helper subsets in labial salivary glands from primary Sjögren’s syndrome patients. *Clin Exp Immunol.* (2012) 169:89–99. 10.1111/j.1365-2249.2012.04606.x 22774983 PMC3406368

[22] Szabo K Papp G Dezso B Zeher M. The histopathology of labial salivary glands in primary Sjögren’s syndrome: focusing on follicular helper T cells in the inflammatory infiltrates. *Mediators Inflamm.* (2014) 2014:631787. 10.1155/2014/631787 25177110 PMC4142299

[23] Blokland SLM Flessa CM van Roon JAG Mavragani CP. Emerging roles for chemokines and cytokines as orchestrators of immunopathology in Sjogren’s syndrome. *Rheumatology.* (2019) key438. 60:3072–87. 10.1093/rheumatology/key438 30838419

[24] Cosorich I McGuire HM Warren J Danta M King C. Ccr9 expressing T helper and T follicular helper cells exhibit site-specific identities during inflammatory disease. *Front Immunol.* (2018) 9:2899. 10.3389/fimmu.2018.02899 30662436 PMC6329311

