# Supplementary material for: Tph Cells Expanded in Primary Sjögren’s Syndrome
Source: Front Med (Lausanne). 2022 Jun 9;9:900349. doi: 10.3389/fmed.2022.900349 (PMC9218540; doi:10.3389/fmed.2022.900349)
Supplement: Supplementary file 1 [file Data_Sheet_1.docx]

**Supplemental Table 1 A detailed flow antibodies strategy**

|  | Tph cells | Tph cells | Plasma cells |
| --- | --- | --- | --- |
| FITC | MHC-II | CD195 (CCR5) |  |
| PerCP/Cy5.5 | CD4 | CD199 (CCR9) |  |
| APC | CD279 (PD-1) | CD279 (PD-1) |  |
| Alexa Fluor 700 |  |  | CD138 |
| APC/Cyanine7 | CCR2 | CD4 |  |
| Brilliant Violet 421 | CD278 (ICOS) |  |  |
| Brilliant Violet 510 | CD45RA | CD45RA |  |
| PE | CD185 (CXCR5) | CD185 (CXCR5) |  |
| PE/Cy7 |  |  | CD19 |


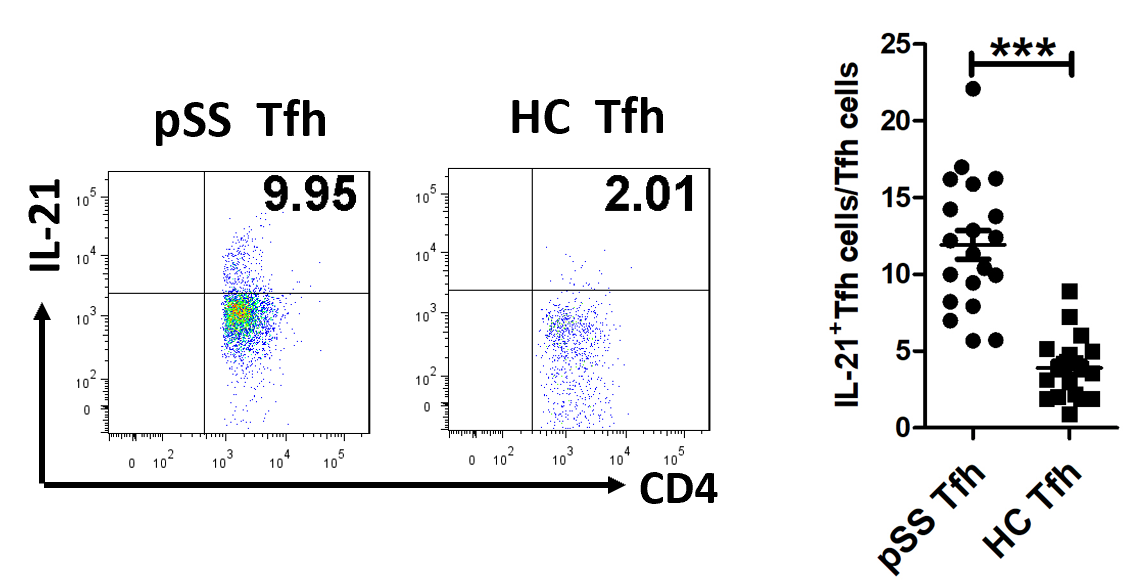


Supplemental Figure 1 The expression of IL-21 was increased in Tfh cells from patients with pSS (n=20), compared to HCs (n=20) (****p*<0.001).
